# Dilated aortic root influences pulmonary artery catheter placement in anesthetized patients

**DOI:** 10.1186/s40981-018-0152-7

**Published:** 2018-02-08

**Authors:** Yuka Miyata, Tsutomu Wada, Tomohiko Hayasaka, Yukio Hayashi

**Affiliations:** Anesthesiology Service, Sakurabashi-Watanabe Hospital, 2-4-32 Umeda, Kita-ku, Osaka, 530-0001 Japan

**Keywords:** Pulmonary artery catheter, Difficult placement, Aortic root

## Abstract

**Purpose:**

The placement of a pulmonary artery catheter sometimes needs long time by observing the pressure wave, and several factors have been reported to hinder the placement. In the present study, we examined whether enlargement of the aortic root is associated with longer time for the placement.

**Method:**

We examined the time required for the catheter placement. The catheter placement time was defined as the duration of time required for the catheter to float from the CVP position to the pulmonary artery. The catheter placement was performed by one experienced physician. We examined the following factors on the catheter placement time: the patient’s age, height, weight, cardiothoracic ratio, tricuspid regurgitation, ejection fraction and the diameter of aortic annulus, sinus of Valsalva, sinotubular junction, and proximal ascending aorta. These diameter values were divided by body surface area (BSA) to equalize among different physical sizes. The data were analyzed by multiple linear regression analysis after univariate analysis.

**Results:**

The univariate analysis showed that ejection fraction and aortic annulus/BSA, sinus of Valsalva/BSA, and sinotubular junction/BSA correlated with the catheter placement time (*P* = 0.079, 0.030, 0.029, and 0.025, respectively). Since the three aortic root values correlated with each other, we chose the sinotubular junction/BSA for the following multivariate analysis, because of the highest *P* value. The multivariate analysis showed that sinotubular junction/BSA had a significant positive association with the placement time (*P* = 0.048).

**Conclusion:**

The present study showed that enlargement of the aortic root is associated with long placement time of the catheter.

A pulmonary artery catheter (PAC) is used for perioperative management in patients undergoing cardiovascular surgery, although the application of the catheter still remained controversial [1–3]. We routinely place the catheter in patients undergoing cardiovascular surgery after induction of anesthesia by monitoring the pressure waveform. The catheter with a balloon on its tip is typically carried by the blood stream to the pulmonary artery readily. However, in some cases, we have experienced longer time to place a catheter by simply observing the pressure wave. In these situations, we may try again under tipping up and down the patient’s bed or need some guidance such as X-ray fluoroscopy or transesophageal echocardiography [4, 5].

The difficulty of the catheter placement may, in part, depend on operator’s skill but to a large extent be due to patient-related factors. These factors may include cardiomegaly, severe tricuspid regurgitation, history of tricuspid ring annuloplasty, low cardiac function, and some anatomical malformation, such as presence of persistent left vena cava [6, 7]. We hypothesized that dilated aortic root and proximal ascending aorta would shift the anatomical position of the right ventricle outflow tract (RVOT) and the pulmonary valve and this change would hinder the tip of the PAC from advancing to the pulmonary artery. In this study, we examined the time required for the catheter placement in adult patients undergoing cardiovascular surgery and determined patient’s factors described above which are associated with the difficult PAC placement.

## Methods

The current study was approved by the institutional review board, and the approved number of subjects in our review board was 100. The informed consent was obtained from all eligible patients, and this study was registered in the UMIN Clinical Trial Registry (UMIN 000027418). This study was conducted from November 2014 to September 2015 at Sakurabashi-Watanabe hospital in Osaka, Japan. We prospectively examined the time required for the PAC placement for 74 adult patients undergoing elective cardiovascular surgery. The patients who had a history of tricuspid ring annuloplasty were excluded. All patients’ electrocardiogram, invasive arterial pressure, oxygen saturation, and end-tidal carbon dioxide were monitored. After the induction of anesthesia with midazolam, fentanyl or remifentanil, and vecuronium, mechanical ventilation was started following tracheal intubation. Anesthesia was maintained with propofol or sevoflurane combined with remifentanil and fentanyl. The PAC (continuous cardiac output/SvO_2_ Catheter 744HF75, Edwards Lifesciences, Irvine, CA, USA) was inserted through the right internal jugular vein by the same experienced physician (YM), who is a certified anesthesiologist of the Japan Society of Anesthesiologists. First, the introducer sheath was placed via the right internal jugular vein in the Trendelenburg position, and then, the PAC started floating through the sheath by monitoring the pressure waveform in the flat position. The PAC was inserted approximately 20 cm, and central venous pressure waveform was confirmed; subsequently, the balloon was inflated with 1.5 ml of air. With inflated balloon, the tip of the catheter floated into the pulmonary artery. The waveform of the pulmonary artery was first observed, followed by inserting the catheter approximately 2–3 cm forward and deflating the balloon. In this position, being confirmed that the tip of the catheter was not wedged into the pulmonary artery, the catheter was locked with the sheath.

The time required for placement of a PAC was measured. The catheter placement time was defined as the duration of time required for the catheter to float from the CVP position through the right heart chambers to the pulmonary artery, that is, the beginning time point was just after the inflation of the balloon to start floating the catheter and the ending time point is the time which we first observed the waveform of the pulmonary artery. If the placement was done within 5 min, we regarded this case as successful. On the other hand, if the placement failed to precede the catheter into the pulmonary artery in 5 min, some guidance such as transesophageal echocardiography or X-ray fluoroscopic system to visualize intracardiac catheter orientation was used.

In this study, we examined the effect of the following factors, which covered the patient’s characteristics, cardiac size and aortic size, and cardiac function, on the catheter placement time: the patient’s age, height, weight, BSA, cardiothoracic ratio (CTR), the diameter of aortic root and proximal ascending aorta, the degree of tricuspid valve regurgitation (TR), and ejection fraction (EF). EF and the degree of TR and CTR were evaluated by transthoracic echocardiography and X-ray examination, respectively, prior to the surgery. The diameter of the aortic root and proximal ascending aorta was obtained by the transesophageal echocardiography in the mid-esophageal long axis view after the induction of anesthesia. These values were measured at the following four points: aortic annulus, sinuses of Valsalva, sinotubular junction, and proximal ascending aorta on the level with right pulmonary artery by another staff physician who was blind to the catheter placement time. These diameters were measured at midsystole from inner edge to inner edge, because the catheter floated through the pulmonary valve during systole. According to the guideline [8], we recorded these values divided by the body surface area (BSA) to equalize among different physical sizes.

Based on first 30 cases, sample size was calculated to be 71 for detecting the diameter of aortic annulus at the significant level of 0.05 with a statistical power of 80%. Thus, we included 74 cases to clear the sample size. Data were expressed as means ± SD or as a median range and interquartile range as appropriate. To predict the difficulty of PAC placement from the factors, a multiple linear regression was conducted. Factors included in multiple linear regression analysis were selected among variables yielding *P* < 0.1 by simple linear regression analysis. All analyses were conducted with SPSS (IBM Corporation, USA) version 14.0. *P* < 0.05 was considered statically significant.

## Results

**Fig. 1:**
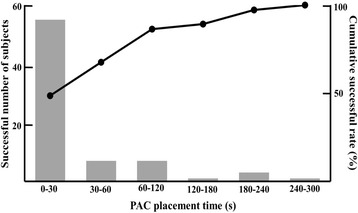
Distribution of the pulmonary artery catheter placement time

The placement of a PAC was successful in all of 74 patients. The patient’s demographic data including patient’s disease targeted to surgery and the catheter placement time is presented in Table 1. The average time to place the catheter was 38 ± 51 s. The distribution of the PAC placement time is presented in Fig. 1. Catheter placement was performed within 5 min in all cases, and 74.3% of the total patients were completed within 30 s. The result of simple and multiple linear regression analysis is shown in Tables 2 and 3, respectively. The univariate analysis showed that EF and the diameters of aortic root/BSA, including aortic annulus, sinuses of Valsalva, and sinotubular junction, significantly correlated with the catheter placement time (*P* = 0.079, 0.030, 0.029, and 0.025, respectively). The three aortic root factors significantly correlated with each other, so we chose the diameter of sinotubular junction/BSA which represented the diameter of aortic root for the following multivariate analysis, because of the highest *P* value in the univariate analysis. The multivariate analysis showed that the diameter of sinotubular junction/BSA had a significant positive association with the time to place a PAC (*P* = 0.048; Table 3).

**Table 1:** Summary of 74 patients

Age (year)	68 ± 12
Height (cm)	159 ± 12
Weight (kg)	57 ± 14
CTR (%)	55 ± 8
LVEF (%)	61 ± 15
Degree of TR	1 (0–2)
Aortic annulus/BSA (mm/m^2^)	14.4 ± 1.8
S Val/BSA (mm/m^2^)	22.0 ± 4.2
STJ/BSA (mm/m^2^)	18.1 ± 3.4
AscAo/BSA (mm/m^2^)	22.0 ± 5.1
Placement time (s)	38 ± 51
Success rate (%)	100
[Disease]	
AS (13) AR (3) AS + AR (4) MR (12) MS (3) CAD (14) DCM (2)	
TAA (10) AAE + AR (5) CAD + AR (1) CAD + MR (2)	
Constrictive Pericarditis (1) Papillary fibroelastoma (1)	
Mixoma (1) Intracardiac tumor (1) CoA (1)	

Data were expressed as means ± SD or as a median range and interquartile range as appropriate

*CTR* cardiothoracic ratio, *LVEF* left ventricular ejection fraction, *TR* tricuspid regurgitation, *BSA* body surface area, *S Val* sinuses of Valsalva, *STJ* sinotubular junction, *AscAo* proximal ascending aorta, *AS* aortic stenosis, *AR* aortic regurgitation, *MS* mitral stenosis, *MR* mitral regurgitation, *CAD* coronary artery disease, *DCM* dilated cardiomyopathy, *TAA* thoracic aorta aneurysm, *AAE* annuloaortic ectasia, *CoA* coarctation of the aorta

**Table 2:** Simple linear regression model of potential predictors of increased pulmonary artery catheter placement time

	Parameter estimation (95% confidence limits)	SE	*P* value
Age (year)	− 0.274 (− 1.23–0.737)	0.493	0.619
Height (cm)	0.653 (− 0.361–1.666)	0.509	0.204
Weight (kg)	0.236 (− 0.613–1.103)	0.435	0.590
CTR (%)	0.461 (− 1.0141–1.936)	0.740	0.535
LVEF (%)	− 0.681 (− 1.442–0.081)	0.382	0.079
Degree of TR	4.43 (− 8.89–17.74)	6.68	0.51
Aortic annulus/BSA (mm/m^2^)	7.29 (0.71–13.85)	3.30	0.03
S Val/BSA (mm/m^2^)	3.09 (0.33–5.86)	1.39	0.029
STJ/BSA (mm/m^2^)	3.95 (0.51–7.39)	1.73	0.025
AscAo/BSA (mm/m^2^)	1.73 (− 0.59–4.04)	1.16	0.142

*CTR* cardiothoracic ratio, *LVEF* left ventricular ejection fraction, *TR* tricuspid regurgitation, *S Val* sinuses of Valsalva, *STJ* sinotubular junction, *AscAo* proximal ascending aorta

**Table 3:** Multivariate linear regression model of potential predictors of increased pulmonary artery placement time

	Parameter estimation (95% confidence limits)	SE	*P* value
LVEF (%)	− 0.475 (− 1.21–0.271)	0.37	0.208
STJ/BSA (mm/m^2^)	3.50 (0.03–6.98)	1.74	0.048

*LVEF* left ventricular ejection fraction, *STJ* sinotubular junction

## Discussion

The essential finding of this study is that the diameter of aortic root is a significant factor to increase the catheter placement time (Tables 2 and 3).

So far, previous clinical researches and case reports have documented several factors to be associated with difficult PAC placement [5, 7, 9, 10]. However, to our knowledge, there have been no clinical studies to demonstrate that dilated aortic root is a significant factor to increase the PAC placement time except one case report [11].

**Fig. 2:**
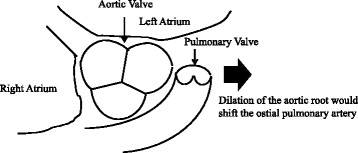
Schematic representation of anatomical relationship between the aortic valve and the pulmonary valve using midesophageal aortic valve short-axis view of transesophageal echocardiography. Dilation of the aortic root and/or the proximal ascending aorta would shift the right ventricle outflow tract and/or the ostial pulmonary artery

The ascending aorta and pulmonary artery are anatomically enclosed by the pericardium and the pulmonary valve is located slightly upper and anterior to the aortic valve (Fig. 2). Thus, we speculate that dilation of the aortic root and/or the proximal ascending aorta would shift the RVOT and/or the ostial pulmonary artery where the PAC has to pass and would change the anatomical position of the RVOT and/or the ostial pulmonary artery, resulting in time-consuming for the PAC placement. Unfortunately, we did not evaluate the anatomical change of the RVOT and/or the ostial pulmonary artery in this study. Thus, we have to acknowledge that we have no definite evidence showing how the passage was affected by the dilated aortic root or ascending aorta. Nevertheless, if our speculation is true, this study would have some clinical suggestions. The PAC placement may be expected to take long time in patients with the dilated aortic root, so, in these cases, we may not hesitate to introduce some guidance such as TEE or X-ray fluoroscopic photographing system.

A previous study demonstrated that as experience increases, the PAC placement time decreases [10]. Thus, our study was designed to perform the PAC placement by only one certified anesthesiologist to avoid the effect of experience. In fact, the PAC placement time did not significantly change during the study period (data are not shown).

One recent clinical study has documented that patient’s preoperative CTR and EF are important factors to affect the PAC placement, when the placement was performed by resident anesthesiologists [7]. On the contrary, the present study did not show that these factors were significant (Table 2). Presumably, the difference in experience of anesthesiologists might cause different results.

We have to discuss potential limitations in our study. First, although we found that the diameter of sinotubular junction/BSA had a significant positive association with the placement time of a PAC (*P* = 0.048; Table 3), the *P* value (0.048) was a little bit smaller than 0.05. Considering that the number of the subjects was 74, we have to acknowledge the possibility of type II error. Second, in this study, we chose the 11 variables, but it might be likely that we overlook another important factor to affect the catheter placement and with that factor, our results would have to be reexamined. Third, our results may reach the statistical significance about the diameter of sinotubular junction/BSA (Tables 2 and 3). However, the results are dependent on statistical analysis, and the clinical significance of our results would be interpreted with caution.

In conclusion, the present data showed that the diameter of aortic root is a significant factor to increase the PAC placement time.
